# Semi-Counterfactual Quantum Bit Commitment Protocol

**DOI:** 10.1038/s41598-020-62893-0

**Published:** 2020-04-16

**Authors:** Yaqi Song, Li Yang

**Affiliations:** 1 National Engineering Laboratory for Public Safety Risk Perception and Control by Big Data, China Academy of Electronics and Information Technology, Beijing, 100041 China; 2 State Key Laboratory of Cryptology, P.O.Box 5159, Beijing, 100878 China; 3 State Key Laboratory of Information Security, Institute of Information Engineering, Chinese Academy of Sciences, Beijing, 100093 China; 4 School of Cyber Security, University of Chinese Academy of Sciences, Beijing, 100049 China

**Keywords:** Quantum information, Computer science

## Abstract

A semi-counterfactual quantum bit commitment (SCQBC) protocol is presented here for the first time, which makes use of counterfactual property. Similar to a counterfactual quantum key distribution scheme, half-photons are not transmitted through the quantum channel in our proposed protocol. In the SCQBC protocol, Bob, the verification party of the quantum bit commitment (QBC), sends the states while Alice, the commitment party, receives. Since Alice cannot receive all the states and entangle the commit bits with the verifier’s registers, it is not subject to Mayers’ and Lo-Chau’s no-go theorem. In addition, a general bit commitment framework can be extracted from the SCQBC scheme, which opens up a new class of cryptographic protocols in counterfactual cryptography.

## Introduction

The bit commitment (BC) scheme is a basic primitive of modern cryptography. The BC concept was first proposed by Blum^1^, and it plays a crucial role in constructions of multi-party secure computation, such as zero-knowledge proof schemes and verified secret shared schemes.

The BC scheme includes two phases, namely, the *commit phase* and *unveil phase*. In the *commit phase*, the commitment party Alice chooses a commit bit *x* and provides a piece of evidence to the verifier Bob. In the *unveil phase*, Alice unveils the value of *x* and Bob checks it. A BC scheme has the following properties. (i) Correctness. If Alice and Bob execute the scheme honestly, Bob obtains the correct commit bit *x* in the *unveil phase*. (ii) Concealing. Bob cannot know the commit bit *x* before the *unveil phase*. (iii) Binding. Alice cannot change the commit bit after the *commit phase*. A BC scheme is unconditionally secure if there is no computational assumption on the attacker’s ability and it satisfies both properties of concealing and binding.

There is at present no classical BC protocol that achieves unconditional security in both concealing and binding. With the development of quantum cryptography, many researchers attempted to construct unconditionally secure QBC. The first QBC scheme was proposed in^2^ but the binding security of the scheme can be attacked by sending entangled states. In 1993, a BCJL scheme was presented^3^ and it was believed provably secure over a period of time until a cheating strategy was put forward by Mayers^4^. Later, Mayers, Lo, and Chau separately presented the no-go theorem and proved that the unconditional secure QBC protocol is impossible^5–7^. Subsequently, a series of studies on extending the framework of the no-go theorem and further proof of the impossibility of QBC has been presented^8–13^. Since no-go theorem was presented, most of QBC protocols cannot realize the unconditional security anymore, especially the binding security.

The later researches focus on exploring QBC with practical security and constructing the QBC protocols evading the no-go theorem type attack. In no-go theorem, Alice prepares a series of entangled states, sends half to Bob and keeps the other half not measured in the commitment phase. In the unveil phase, Alice can apply a local unitary transformation on the remaining half qubits to rotate her commitment. It can be seen that the requirement of the no-go theorem type attack is hardly realized in practice. There are several QBC protocols proposed under the physical hypothesis, such as the bounded-quantum-storage model^14,15^, noisy-storage model^16–18^, technological limitations on non-demolition measurements^19^ and relativistic QBC protocols^20–22^. In addition, since the algorithm needs at least *O*(2^2*n*^) size of memory space to store the matrix of the unitary transformation^23^, where *n* is the security parameter of the QBC, Song and Yang^24^ constructed a practical QBC protocol with physical security. On the other hand, although the correctness of the no-go theorem is not doubted, the framework of the theorem may not cover all the types of QBC protocols^25^. People try to construct QBC protocols to resist the attack presented by the no-go theorem^26–30^.

We are inspired by counterfactual quantum cryptography^31^ and try to construct a QBC protocol immune to no-go theorem type attack based on the counterfactual property. The counterfactual quantum key distribution protocol (N09) was presented by Tae-Gon Noh, in which the particle carrying secret information is not in fact transmitted through the quantum channel. The counterfactual property makes that the receiver has no idea about the information carried by the sender’s local particle as long as the responses of the detectors are not disclosed. Let the verifier Bob to be the sender of the counterfactual system and the commitment party Alice to be the receiver. It seems that there is some information gap caused by Bob’s local particles, which leads that Alice cannot dishonestly change the commitment without detection. In this paper, we show that it is possible to build a QBC protocol based on the counterfactual property, called semi-counterfactual quantum bit commitment (SCQBC), which can resist the existing no-go theorem type attack. Then we analyze the general cheating strategies, such as intercept attack and intercept/resend attack, and find there is no effective attack to break the concealing and binding security. Quite interestingly, a general BC model is extracted from SCQBC, which may provide a new idea to achieve unconditionally secure BC protocols.

## Results

### Semi-counterfactual quantum bit commitment

**Figure 1:**
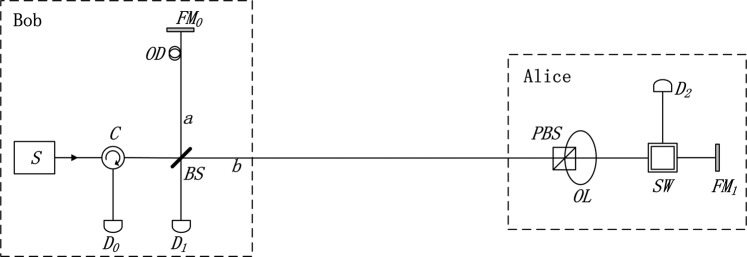
Architecture of N09 and SCQBC protocols. The setup is a modification based on a Michelson-type interferometer. The single-photon source *S* emits an optical pulse containing only one photon. The pulse is then transmitted through the optical circulator *C* and split into two pulses by the beam splitter *BS*. The two light paths *a* and *b* are the arms of the Michelson-type interferometer, and the length of the path *a* is adjusted by an optical delay *OD*. The pulse transmitted through path *a* is reflected by the Faraday mirror *FM*_0_ and back to the *BS*. The pulse transmitted through path *b* travels to Bob’s site. If the pulse is horizontally polarized, it passes through the polarizing beam splitter *PBS*, or it is reflected by *PBS* and passes through the optical loop *OL*. The arrival time to the optical switch *SW* of the different polarized pulses is different. Only when the *SW* is controlled in the correct time will the pulse reach the detector *D*_2_. Otherwise, the pulse will be reflected by *FM*_1_ and return to Alice’s site. The back-pulse from path *b* and the pulse from path *a* are combined at the *BS* and interfered to lead the detector *D*_0_ to click.

Figure 1 illustrates the diagram of the experimental implementation of N09^31^. The aim of QKD protocols is to share the secret keys between the two parties Alice and Bob, which prevent giving out information from the eavesdroppers. Then, the positions and functions of Alice and Bob are equivalent, swapping of the positions is allowed. No matter who is the sender or the receiver, N09 protocol can be executed correctly. In QBC protocols, Alice is the commitment party while Bob is the verification party. To satisfy both binding (i.e. Alice cannot change the commitment bit *x* after the *commit phase*) and concealing (i.e. Bob cannot know *x* before the *unveil phase*), Bob is designed as the sender and Alice as the receiver. The experimental implementation of SCQBC is shown in Fig. 1 and described as follows.

The single-photon source *S* emits an optical pulse containing only one photon. The polarization of the single-photon state is chosen by Bob, the horizontal polarization |*H*〉 representing the bit value “0” while vertical polarization |*V*〉 representing the bit value “1”. The pulse then is transmitted through the optical circulator *C*. The circulator *C* is in clockwise direction, through which the left light transmits to the right optical path and the right light goes to the detector *D*_0_ in the below optical path. When Bob sends a horizontal polarized state, it split into the horizontal polarization in mode *a* and mode *b* by the beam splitter *BS*, denoted as |*H*〉_*a*_ and |*H*〉_*b*_, respectively. |*H*〉_*a*_ passes through the optical path only in Bob’s local site. Then it is rotated by the Faraday mirror *FM*_0_ to |*V*〉_*a*_ and reflected back. For the other part |*H*〉_*b*_, it passes through the *PBS* and goes directly to *SW*. If Alice does nothing with *SW*, |*H*〉_*b*_ goes to the Faraday mirror *FM*_1_, then it is rotated to |*V*〉_*b*_ and reflected back to *SW*. |*V*〉_*b*_ then is reflected by *PBS*, passes through the optical loop *OL* and goes through the quantum channel. Removing the detector *D*_2_ and optical switch *SW*, the remaining devices construct a conventional structure of Michelson interferometer. Through adjusting the two arms accurately, |*V*〉_*a*_ and |*V*〉_*b*_ arrive at *BS* simultaneously and the interference is detected by *D*_0_. When Bob sends a vertical polarized state |*V*〉, the difference with |*H*〉 is that |*V*〉_*b*_ is reflected by *PBS*, goes through the optical loop *OL*. The different polarizations arrive at the switch *SW* in different time. Actually, Alice also chooses one of the two polarizations and blocks some of the pulses using the setup according to her bits. If Alice chooses bit “0”, corresponding to polarization |*H*〉, she will block the horizontal polarization in the optical path *b*. If Alice chooses bit “1”, she will block the vertical polarization in the optical path *b*. She can effectively switch the polarization state to the detector *D*_2_ by accurately controlling the switch time. When Alice’s bit is different with Bob’s, she controls the *SW* at the incorrect time, which is equivalent to that she does nothing, to lead the setup to form a Michelson interferometer and the detector *D*_0_ clicks. When Alice’s bit is the same as Bob’s, she controls the *SW* at the suitable time. In this case, the interference is destroyed and there are three occasions for the single photon. (i) Detector *D*_0_ clicks. The photon travels via path *a* and then is reflected by the *BS* to the detector *D*_0_. (ii) Detector *D*_1_ clicks. The photon travels via path *a* and then passes through the *BS* to the detector *D*_1_. (iii) Detector *D*_2_ clicks. The photon travels via path *b* and is controlled by the *SW* to the detector *D*_2_.

In N09 protocol, when the detector *D*_0_ or *D*_2_ clicks, Alice and Bob announce the detected and initial polarization states. When the detector *D*_1_ clicks, Alice compares the detected polarization with the initial polarization: if they are inconsistent, she announces the detection; otherwise, she keeps secret. Only the clicks of *D*_1_ are the generation of the secret keys, in which situation the information carriers are only transmitted in Bob’s site rather than the quantum channel. We find that if Alice and Bob keep their measurement results secret, the information obtained by them is not asymmetric. Inspired by the counterfactual property, we proposed a SCQBC protocol. Then tease out the relationship between the two parties’ bits and the response of the detectors: (i) *D*_0_ clicks. Whether the bits chosen by Alice and Bob are consistent or not, the event that the detector *D*_0_ clicks can happen. Although Bob knows the detection, he never knows Alice’s bit. (ii) *D*_1_ clicks. Only when the chosen bits are inconsistent, it is possible that *D*_1_ clicks. In this case, Bob knows Alice’s bit but the information carrier is never transmitted through the quantum channel, which is counterfactual. (iii) *D*_2_ clicks. Only when the chosen bits are inconsistent, it is possible that *D*_2_ clicks. Ideally, *S* is a single-photon source and there is only one photon in the system. If Bob’s detectors do not click, he knows *D*_2_ clicks and what Alice’s bit is. In addition, Alice also knows Bob’s bit. A secure QBC protocol needs to be both concealing and binding. The events that *D*_0_ clicks can guarantee the concealing, in which Bob cannot know Alice’s bits. The events that *D*_1_ or *D*_2_ clicks can be the evidence of the commitment and guarantee the binding, in which Bob knows Alice’s bit exactly and Alice’s change of these bits can be easily detected.

Before the SCQBC protocol, there are three time parameters to be determined, as follows: Δ*t*_0_, the time that a photon spends from the source *S* through the polarizing beam splitter *PBS* to the optical switch *SW*, where the optical path of the photon is *S* → *C* → *BS* → *PBS* → *SW* → *D*_2_; Δ*t*_1_: the time that a photon spends from the source *S* through the optical loop *OL* to the optical switch *SW*, where the optical path is *S* → *C* → *BS* → *OL* → *SW* → *D*_2_; Δ*t*_2_, the time that a photon spends from the source *S*, reflected by *FM*_1_ to Bob’s site again, where the optical path is *S* → *C* → *BS* → *PBS*(*OL*) → *FM*_1_ → *OL*(*PBS*) → *BS* → *D*_1_(*D*_0_). Note that Δ*t*_2_ is also the time that a photon spends from the source *S*, reflected by *FM*_0_ to Bob’s detectors, where the optical path is *S* → *C* → *BS* → *FM*_0_ → *BS* → *D*_1_(*D*_0_). Alice and Bob perform tests to measure the time parameters by sending and detecting some quantum states. To be specific, Bob sends a series of states |*H*〉 or |*V*〉 to Alice and tells her what the states are before sending. Then, Alice tries to control the optical switch *SW* in proper time to make detector *D*_0_, *D*_1_, *D*_2_ click, respectively. Through this test, three time parameters can be measured.

### Protocol 1 Semi-counterfactual quantum bit commitment

#### Commit phase

1. *Alice and Bob set up devices according to Fig*. 1, *where the beam splitter BS is a standard half-transparent and half-reflecting mirror. They share four security parameters m, n, k and N*.
2. *Alice generates m bit strings randomly and uniformly with the length of N. Each sequence is represented as* ${a}^{(i)}\equiv ({a}_{1}^{(i)}{a}_{2}^{(i)}\ldots {a}_{N}^{(i)})\in {\{0,1\}}^{N}$, $i\mathrm{=1,}\,\mathrm{2,}\ldots ,m$.
3. *Bob generates m bit strings randomly and uniformly with the length of N. Each sequence is represented as* ${b}^{(i)}\equiv ({b}_{1}^{(i)}{b}_{2}^{(i)}\ldots {b}_{N}^{(i)})\in {\{0,1\}}^{N}$.
4. *Alice and Bob decide on a series of time instants* ${t}_{1}^{(i)},{t}_{2}^{(i)},\ldots ,{t}_{N}^{(i)}$. *Bob sends* $|{\Psi }_{{b}_{j}^{(i)}}\rangle$ *at the time* ${t}_{j}^{(i)}$, *while Alice controls the switch with bit* ${a}_{j}^{(i)}$. *When* ${a}_{j}^{(i)}\mathrm{=0}$, *she controls SW at the time* ${t}_{j}^{(i)}+\Delta {t}_{0}$; *when* ${a}_{j}^{(i)}=1$, she controls SW at the time ${t}_{j}^{(i)}+\Delta {t}_{1}$. $|{\Psi }_{0}\rangle =|H\rangle$ *and* $|{\Psi }_{1}\rangle =|V\rangle$ *represent the horizontally polarized state and vertically polarized state, respectively*.
5. *Alice and Bob record the response of the detector* *D*_2_, *D*_0_, *and* *D*_1_ *as* *α* ∈ {0, 1}, *β*_0_ ∈ {0, 1}, *and* *β*_1_ ∈ {0, 1}, *respectively*. *α*, *β*_0_, *and* *β*_1_ = 0 *denote that there is no click in the related detector*. *α*, *β*_0_, *and* *β*_1_ = 1 *denote the related detector clicks. Note that as long as the detectors do not click in the correct time they record the result* “0.” *For example, if Bob’s detectors* *D*_0_ and *D*_1_ *have not clicked until* ${t}_{j}^{(i)}+\Delta {t}_{2}$, *he records* *β*_0_ = *β*_1_ = 0.
6. *For each sequence of states, Alice verifies whether the detection of* *D*_2_ *is approximately* *N*/4. *If the proportion is incongruent, she believes that Bob cheated and she aborts. For each sequence of states, Bob selects k bits to be verified, then asks Alice to publish the corresponding values of* ${a}_{j}^{(i)}$ *and the response of the detector* *D*_2_.
7. *Alice chooses a random bit* *x* ∈ {0, 1} *as her committed bit. Then she selects n bits from the N − k bits except the verified bits, which satisfies* ${a}_{1}^{(i)}\oplus {a}_{2}^{(i)}\oplus \ldots \oplus {a}_{n}^{(i)}=x$.

#### Unveil phase

1. *Alice reveals the value of x, m sequences of* $({a}_{1}^{(i)}{a}_{2}^{(i)}\ldots {a}_{N}^{(i)})$, $i=\mathrm{1,}\,\mathrm{2,}\ldots ,m$, *the response of the detector* *D*_2_ *and the index of n bits of each sequence to Bob*.
2. *Bob verifies whether* ${a}_{1}^{(i)}\oplus {a}_{2}^{(i)}\oplus \ldots \oplus {a}_{n}^{(i)}=x$, *and the response of all the detectors agree with the state* $|{\Psi }_{{b}_{j}^{(i)}}\rangle$. *If the consistency of all of the data holds, he admits Alice’s commitment value as x; otherwise, if any of the data are inconsistent, he concludes that Alice cheated*.

### Correctness

The commit bit $x={a}_{1}^{(i)}\oplus {a}_{2}^{(i)}\oplus \ldots \oplus {a}_{n}^{(i)}$. It is oblivious that when both two parities execute the SCQBC honestly, all of the devices used are ideal and perfect, Bob can obtain the correct commit bit.

In a secure BC protocol, Alice chooses a commit bit *x* and gives a piece of evidence to Bob. The key point is that Bob cannot recover the commit bit by the evidence and Alice cannot change the commit bit without detection due to the limitation of the evidence. For SCQBC protocol, the evidence is the data detected by *D*_1_ and *D*_2_, in which situation that Bob knows exactly that ${a}_{j}^{(i)}={b}_{j}^{(i)}$. When the detector *D*_1_ clicks, the particle generated by Bob is not transmitted through the quantum channel but carries the information of ${a}_{j}^{(i)}={b}_{j}^{(i)}$, which satisfies the counterfactual property. When the detector *D*_2_ clicks, the particle generated by Bob is transmitted through the quantum channel to Alice’s side with the information of ${a}_{j}^{(i)}={b}_{j}^{(i)}$, which is a factual phenomenon. Above all, some particles carrying evidence information are transmitted in the channel while others are not. It is the reason why Protocol 1 is defined as the semi-counterfactual quantum bit commitment rather than counterfactual protocol.

## Security analysis

### Basic ideas

A bit comparison function with two participants can be realized by the counterfactual setup. In the bit-comparison function module, Alice and Bob first randomly choose a bit, respectively, then compare the values though sending and detecting the photons using the setup in Fig. 1. There are three results of comparison: (i) Both participants can confirm the bit chosen by each other, and he (she) also knows that the other one have confirmed his (her) bit; (ii) Bob confirms Alice’s bit while Alice knows nothing; (iii) they both know nothing. Suppose there are two critical parameters *p* and *q*, where *p* is the ratio that Bob confirms the value of Alice’s bit among *n* bits, *q* is the ratio that Alice knows that Bob confirms her bit. Then the parameters satisfy 0 ≤ *q* < *p* < 1 may achieve a secure BC protocol. The specific explanation is as follows.

In the BC protocol, Bob should have a piece of evidence to detect whether Alice cheats. *p* > 0 guarantees the information content of the evidence not to be zero. Since concealing security requires that Bob cannot obtain the committed bit before the *unveil phase*, Bob should not know all of Alice’s bits correctly, i.e., *p* < 1. Then, 0 < *p* < 1, and a BC protocol can satisfy the concealing security by choosing appropriate security parameter *n*. If Alice cheats and tries to alter one bit in the *unveil phase*, her best choice is to select a bit that she cannot distinguish whether Bob confirms rather than the bit in the first kind. For each sequence, there are approximately (1 − *p*)*n* qubits that Bob cannot judge. If *p* = *q*, Alice can accurately alter the bit in part that Bob really does not know without detection. If *q* < *p*, the range of bits that can be altered by Alice is larger than that Bob cannot distinguish, and her attack may be caught. Therefore, *q* < *p* is the necessary condition of the binding security.

In the SCQBC protocol, parameters *p* and *q* depends on the response of the detectors. The concrete analysis is as follows. Bob sends single-photon states |*H*〉 and |*V*〉 representing the bit value “0” and “1.” After transferring through the beam splitter *BS*, the initial states become

1 $$\begin{array}{rcl}|{\phi }_{0}\rangle  & = & \sqrt{t}|0{\rangle }_{a}|H{\rangle }_{b}+i\sqrt{r}|H{\rangle }_{a}|0{\rangle }_{b},\\ |{\phi }_{1}\rangle  &  & \sqrt{t}|0{\rangle }_{a}|V{\rangle }_{b}+i\sqrt{r}|V{\rangle }_{a}|0{\rangle }_{b},\end{array}$$

where *a* and *b* represent the mode towards Bob’s Faraday mirror *FM*_0_ and the mode towards Bob’s site, respectively, seen in Fig. 1. *t* and *r* are the transmissivity and reflectivity of the *BS*, respectively. Both $|{\phi }_{0}\rangle$ and $|{\phi }_{0}\rangle$ can be denoted a Fock state $|\phi \rangle =\sqrt{t}\mathrm{|0}{\rangle }_{a}\mathrm{|1}{\rangle }_{b}+i\sqrt{r}\mathrm{|1}{\rangle }_{a}\mathrm{|0}{\rangle }_{b}$.

When ${a}_{j}^{(i)}={b}_{j}^{(i)}$, the state $|\phi \rangle$ collapses to one of the two states, $\mathrm{|0}{\rangle }_{a}\mathrm{|1}{\rangle }_{b}$ or $\mathrm{|1}{\rangle }_{a}\mathrm{|0}{\rangle }_{b}$, due to Alice’s measurement with probability *t* and *r*, respectively. The state $\mathrm{|1}{\rangle }_{a}\mathrm{|0}{\rangle }_{b}$ goes past the *BS* again and becomes $\sqrt{t}|0{\rangle }_{0}|1{\rangle }_{1}+i\sqrt{r}|1{\rangle }_{0}|0{\rangle }_{1}$, where the subscripts 0 and 1 represent the path containing *D*_0_ and *D*_1_, respectively. Therefore, the total probability that *D*_0_ detects the photon is *r*^2^ and the probability that *D*_1_ detects the photon is *rt*. When ${a}_{j}^{(i)}\ne {b}_{j}^{(i)}$, one of the paths introduces the *π* phase and the initial state becomes $\sqrt{t}|0{\rangle }_{a}|1{\rangle }_{b}-i\sqrt{r}|1{\rangle }_{a}|0{\rangle }_{b}$. Then, the state passes the *BS* again and becomes

2 $$\begin{array}{ll} & \sqrt{t}|0{\rangle }_{a}|1{\rangle }_{b}-i\sqrt{r}|1{\rangle }_{a}|0{\rangle }_{b}\\ \mathop{\longrightarrow }\limits^{BS} & \sqrt{t}(\sqrt{t}|1{\rangle }_{0}|0{\rangle }_{1}+i\sqrt{r}|0{\rangle }_{0}|1{\rangle }_{1})-i\sqrt{r}(\sqrt{t}|0{\rangle }_{0}|1{\rangle }_{1}+i\sqrt{r}|1{\rangle }_{0}|0{\rangle }_{1})\\ = & t|1{\rangle }_{0}|0{\rangle }_{1}+i\sqrt{rt}|0{\rangle }_{0}|1{\rangle }_{1}-i\sqrt{rt}|0{\rangle }_{0}|1{\rangle }_{1}+r|1{\rangle }_{0}|0{\rangle }_{1}\\ = & |1{\rangle }_{0}|0{\rangle }_{1}.\end{array}$$

It can be seen that when ${a}_{j}^{(i)}\ne {b}_{j}^{(i)}$ the photon is detected by *D*_0_ with a probability 100%.

The detection probabilities of each detector are listed in Table 1. When the detector *D*_1_ or *D*_2_ clicks (detector *D*_0_ does not click), Bob confirms that Alice’s bit is the same as his. It can be seen that

3 $$p=p({a}_{j}^{(i)}={b}_{j}^{(i)},{\beta }_{1j}^{(i)}=1)+p({a}_{j}^{(i)}={b}_{j}^{(i)},{\alpha }_{j}^{(i)}=1)=\frac{1}{2}(rt+t)=\frac{3}{8}.$$

**Table 1:** Detection probability of each detector. *r* and *t* are the reflectivity and transmissivity of the beam splitter *BS*, respectively.

	${{\boldsymbol{a}}}_{{\boldsymbol{j}}}^{({\boldsymbol{i}})}\ne {{\boldsymbol{b}}}_{{\boldsymbol{j}}}^{({\boldsymbol{i}})}$	${{\boldsymbol{a}}}_{{\boldsymbol{j}}}^{({\boldsymbol{i}})}={{\boldsymbol{b}}}_{{\boldsymbol{j}}}^{({\boldsymbol{i}})}$
${\beta }_{0j}^{(i)}\mathrm{=1}$	1	*r*^2^
${\beta }_{1j}^{(i)}\mathrm{=1}$	0	*rt*
${\alpha }_{j}^{(i)}\mathrm{=1}$	0	*t*

When *D*_0_ clicks, it can be seen that $p({a}_{j}^{(i)}\ne {b}_{j}^{(i)}) > p({a}_{j}^{(i)}={b}_{j}^{(i)})$. Although Bob cannot confirm the value of ${a}_{j}^{(i)}$, he can guess ${a}_{j}^{(i)}\ne {b}_{j}^{(i)}$ with a correct probability of $p({a}_{j}^{(i)}\ne {b}_{j}^{(i)},{\beta }_{0j}^{(i)}=1)$, where

4 $$p({a}_{j}^{(i)}\ne {b}_{j}^{(i)}|{\beta }_{0j}^{(i)}=1)=\frac{1}{1+{r}^{2}}=\frac{4}{5},$$

and

5 $$p({a}_{j}^{(i)}\ne {b}_{j}^{(i)},{\beta }_{0j}^{(i)}=1)=p({a}_{j}^{(i)}\ne {b}_{j}^{(i)}|{\beta }_{0j}^{(i)}=1)\times p({\beta }_{0j}^{(i)}=1)=\frac{1}{2}.$$

Then, the probability that Bob guesses Alice’s bit ${a}_{j}^{(i)}$ correctly is

6 $$p{\prime} =p+p({a}_{j}^{(i)}\ne {b}_{j}^{(i)},{\beta }_{0j}^{(i)}=1)=7/8.$$

When the detector *D*_2_ clicks, Alice confirms Bob has obtained her bit. Therefore,

7 $$q=\frac{1}{2}t=1/4.$$

#### Binding security

If Alice tries to attack the binding of the BC framework, her general strategy is to alter odd bits for each sequence in the *unveil phase*. For each sequence which is in connection with the commitment *x*, she can distinguish that there are approximately *qn* bits confirmed by Bob. Alice’s optimal strategy is to alter one bit in the range of the other (1 − *q*)*n* bits. Among the (1 − *q*)*n* bits, only (1 − *p*)*n* bits are not known by Bob. Therefore, the probability that Alice alters one bit without detection in one sequence is

8 $$p(Aalter)=\frac{(1-p)n}{(1-q)n}=\frac{1-p}{1-q}=5/6.$$

For an *m*-sequence BC protocol, one of the cheating strategies for Alice is to commit “0” with the number of *m*/2 while committing “1” with the number of *m*/2 in the *commit phase*, and changing half of the sequences in the *unveil phase*. Then, the probability of Alice changing the committed bit without detection is *p*(*Aatler*)^*m*/2^. Since *p*(*Aatler*) < 1, *p*(*Aatler*)^*m*/2^ can be exponentially small and the protocol can satisfy the binding security by choosing appropriate security parameter *m*.

#### Concealing security

In the bit-comparison function module, there is a percentage *p* that Bob confirms the values of Alice’s bits. For those bits he cannot confirm, he may just guess. Then, he has a larger probability *p*′ > *p* to guess the value correctly. For a sequence of qubits, Bob makes sure the commitment value has a probability of *p*′^*n*^. Given *m* qubit strings, the probability that Bob has no idea about the commitment value is (1 − *p*′^*n*^)^*m*^. Defining *ε* as the probability that Bob ascertains the commitment value,

9 $$\varepsilon \equiv 1-{(1-p{\prime} n)}^{m}.$$

If Bob does not confirm the commitment value from the protocol, he just guesses with a probability 1/2. Therefore, the probability that Bob obtains the right commitment value is

10 $$p(Bknows)=\varepsilon +\frac{1-\varepsilon }{2}=\frac{1}{2}+\frac{\varepsilon }{2}.$$

Then, the advantage of Bob breaking the concealing security is

11 $$|p(Bknows)-\frac{1}{2}|=|\frac{1}{2}+\frac{\varepsilon }{2}-\frac{1}{2}|=\frac{\varepsilon }{2}=\frac{1-{(1-p{\prime} n)}^{m}}{2}.$$

According to Binomial Theorem,

12 $$(1-p{\prime} n)m=1-mp{\prime} n+\frac{m(m-1)}{2}p{\prime} 2n-\ldots +{C}_{m}^{i}(-p{\prime} n)i+\ldots +(-p{\prime} n)m.$$

Since 0 < *p*′ < 1 and the parameters *m* and *n* are usually larger than 100, *p*′^*n*^ is exponentially small, then the higher order terms of *p*′^*n*^ is going to be negative. Therefore, ${\mathrm{(1}-p{\prime} n)}^{m}$ ≃ $1-mp{\prime} n$. Then

13 $$|p(Bknows)-\frac{1}{2}|\simeq \frac{1-(1-mp{\prime} n)}{2}=\frac{mp{\prime} n}{2}.$$

$|p(Bknows)-\frac{1}{2}|$ can be exponentially small and the protocol can satisfy the concealing security by choosing appropriate security parameters *m* and *n*.

## Security of SCQBC against general attacks

In this subsection, we analyze the possible attacks for the protocol. A cheating Alice tries to change the committed bit in the *unveil phase* without being discovered, while a cheating Bob tries to learn more information of the committed bit during the *commit phase*. The schematic of SCQBC is relatively simple and there exist only a few attacks.

### Concealing against Bob’s cheating strategies

In SCQBC, the operations of Bob is sending and detecting the photons. Bob controls the emission device of SCQBC. If he wants to cheat and obtain more information about the committed bit, his strategy is either changing an optimal device or sending the illegal states.

#### Illegal-state attack

If Bob sends illegal single-photon states with different polarizations, such as |+〉 or |−〉, it just influences the photons transmitted or reflected by *PBS*. Moreover, it can never make Bob learn more information, which is an ineffective attack.

Bob may attack by sending illegal multi-photon states. When multiple photons are transferred in the scheme, the number of photons detected by *D*_2_ will be larger than *n*/4. In Step 6 of SCQBC, Alice verifies the detection of *D*_2_ and this attack can be discovered by the check.

For another illegal-state attack, Bob can prepare entangled states and send one of the particles to Alice. In general, sending entangled states leads the sender to obtain more information about the measurement of the receiver in other QBC protocols. However, the data are encoded with orthogonal single-photon states in our SCQBC protocol. Sending entangled states cannot make Bob obtain more information than single-photon states. However, the measurement is just single-photon detection without choosing the basis. Bob, however, can easily distinguish whether Alice detects the photon because he sends single-photon states and the state is either detected by him or by Alice. There is no secret measurement basis or secret measurement results that must be stolen by entanglement. Therefore, preparing entangled states is a superfluous cheating strategy and there is no advantage for Bob.

#### Optimal-device attack

It can be seen from Eq. 11 that the advantage of a cheating Bob is influenced by the parameter *p*′. According to Eq. 6, the parameter *p*′ is determined by the reflectivity of the beam splitter *BS*. A cheating Bob may not be using a standard half-transparent and half-reflecting mirror in the protocol. Assuming that the transmissivity of the illegal *BS* is *t*′, then clicks of *D*_2_ are approximately *t*′/2. Different beam splitters lead different clicks of *D*_2_. This attack can also be detected by the check in Step 6 of SCQBC.

**Figure 2:**
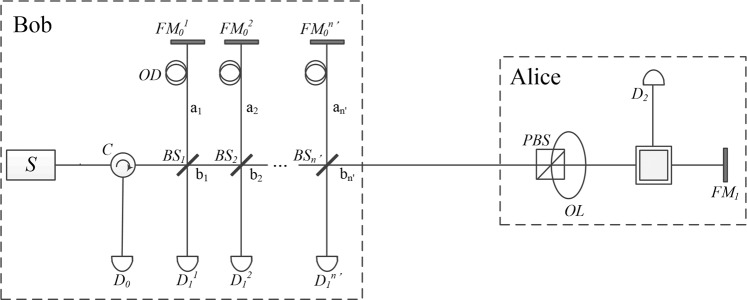
Schematic of Bob’s optimal-device attack, where the number of the *BS* in Bob’s side is *n*′ and the transmissivity of each *BS* is $t{=2}^{-\mathrm{1/}n{\prime} }$.

There is a particular optimal-device attack for Bob. That is, he replaces the standard half-transparent and half-reflecting mirror with *n*′ *BS*, as seen in Fig. 2, where the architecture was fist proposed to increase the efficiency of counterfactual QKD in ref. ^32^. Clearly, the probability that *D*_2_ clicks is ${t}^{n{\prime} }$. In order to make the attack unfound by Alice, the transmissivity of each *BS* of Bob is taken as $t={2}^{-\mathrm{1/}n{\prime} }$. In this attack, if Alice chooses a different bit from that of Bob’s, *i.e*. ${a}_{j}^{(i)}\ne {b}_{j}^{(i)}$, so that she reflects the photon back, it will make the detector *D*_0_ click with certainty. On the other hand, when ${a}_{j}^{(i)}={b}_{j}^{(i)}$ so that Alice blocks the path in her site with the detector *D*_2_, there are three possibilities:

- The photon passes all the *n*′ *BS* and makes *D*_2_ click, which occurs with probability ${P}_{2}={t}^{n{\prime} }\mathrm{=1/2}$.

- The photon is detected by the detector *D*_0_, which occurs with probability *P*_0_ = *rr* + *trrt* + *ttrrtt* + … + (*r*^2^) [*t*^2^^(*n*′-1)^], where *r* = 1 − *t* is the reflectivity of each *BS*.

- The photon is detected by any of the *D*_1_ detectors. The total probability is ${P}_{1}=1-{P}_{2}-{P}_{0}$.

Since Alice has the equal probability 1/2 to choose either the same bit or a different bit, then *D*_0_ clicks with probability

14 $$\begin{array}{cc}Pro{b}_{0}\, & =\frac{1}{2}+\frac{{P}_{0}}{2}\\  & =\frac{1}{2}+\frac{1}{2}{r}^{2}[1+{t}^{2}+{t}^{4}+....+{t}^{2(n{\rm{{\prime} }}-1)}]\\  & =\frac{1}{2}+\frac{{(1-t)}^{2}}{2}\cdot \frac{1-{t}^{2n{\rm{{\prime} }}}}{1-{t}^{2}}\\  & =\frac{1}{2}+\frac{(1-{t}^{2n{\rm{{\prime} }}})}{2}\cdot \frac{1-t}{1+t},\end{array}$$

*D*_1_ clicks with probability

15 $$Pro{b}_{1}=\frac{{P}_{1}}{2}=\frac{1}{4}-\frac{(1-{t}^{2n{\prime} })}{2}\cdot \frac{1-t}{1+t},$$

*D*_2_ clicks with probability $Pro{b}_{2}=({P}_{2})/2=1/4$. Therefore, Bob can always pass the security in step 6 of the protocol.

In this attack, Bob assumes ${a}_{j}^{(i)}={b}_{j}^{(i)}$ when *D*_0_ clicks, otherwise he assumes ${a}_{j}^{(i)}\ne {b}_{j}^{(i)}$. We can find that the error rate that Bob makes a wrong guess on Alice’s bit is

16 $$err=Pro{b}_{0}-\frac{1}{2}=\frac{(1-{t}^{2n{\prime} })}{2}\cdot \frac{1-t}{1+t},$$

**Figure 3:**
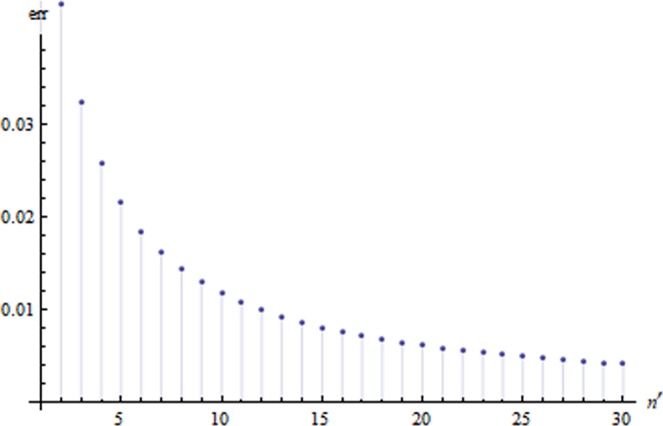
The error rate that Bob makes a wrong guess on each of Alice’s bit.

The relation of the error rate and the number of the *BS* is shown in Fig. 3.

In theory, for a finite *N* value chosen in SCQBC protocol, Bob can find a sufficiently high yet finite value of *n*′, so that *err* ≪ 1/*N*. That is, with the above strategy he can learn all the *N* bits that Alice chosen in step 2 with less than 1 bit of error in average. Then Bob can eventually learn Alice’s committed bit with a non-trivial probability. However, the attack is limited by two techniques and there exist the strategies against the attack in corresponding of the two limitations.

- The manufacturing technique of the beam splitter. In the attack, the accuracy requirement of the transmissivity of each *BS* is very high, which is $t={2}^{-\mathrm{1/}n{\prime} }$. Even though the accuracy meets the requirement of the attack, there is a physical extremes of the transmissivity, which leads a finite *n*′ actually. Give the upper limit of the transmissivity and *n*′, there exists a finite value *N* to guarantee the concealing of SCQBC protocol.

- The technique of controlling the length of the arms in multiple interferometers. In the preparation of SCQBC, Bob and Alice should adjust the optical path *a* and *b* to make the equipment as an interferometer when ${a}_{j}^{(i)}\ne {b}_{j}^{(i)}$. But in the attack, Bob needs to adjust the optical path *a*_1_ and *b*_1_, *a*_2_ and *b*_2_,…, *a*_*n*′_ and *b*_*n*′_, respectively. As long as setting a certain number of photons in the adjustment process, Bob can hardly use a few photons to adjust *n*′ arms and not be detected by Alice.

### Alice’s cheating strategies

All of the cheating strategies can be divided into two categories, i.e. entangled attack and non-entangled attack. For Alice with no ability of entanglement, she is the receiver in SCQBC protocol and there are few available attacks. Because Alice cannot change the initial states, she can only employ a new set of measuring equipment or cheat by returning the illegal states, i.e., intercept attack and intercept/resend attack. However, these two attacks always lead to different click ratios of Bob’s detectors and can be discovered by Bob. The second is a cheating Alice with the ability of entanglement. The most general and famous attack is the no-go theorem attack. However, Alice has neither enough particles nor information to perform this attack. The specific analysis follows.

#### Intercept attack

For the states transferred to Alice in the legal SCQBC protocol, some are detected by Alice while others are reflected by *FM*_1_ to Bob. A cheating Alice who performs an intercept attack can choose to detect all the states and reflect none to Bob; that is, she controls the optical switch *SW* both at the times ${t}_{j}^{(i)}+\Delta {t}_{0}$ and ${t}_{j}^{(i)}+\Delta {t}_{1}$. Then, once the initial states are transmitted through the beam splitter *BS*, the detector *D*_2_ clicks and the probability of that is *t* = 1/2. When the detector *D*_2_ clicks, Alice knows that Bob confirms her bit with *q* = *t* = 1/2. According to Eq. 8, a larger *q* makes Alice a higher probability of attack. However, since the photons are either detected by Alice or Bob, Bob can easily discover the different detection ratios and discover the cheating Alice. Therefore, Alice should select only a few of the photons to intercept.

Assuming Alice selects *n*_0_ bits to intercept, for these *n*_0_ photons she controls the optical switch *SW* both at the times ${t}_{j}^{(i)}+\Delta {t}_{0}$ and ${t}_{j}^{(i)}+\Delta {t}_{1}$. Then, the number of photons detected by *D*_2_ is *tn*_0_, the number of photons detected by *D*_0_ is *r*^2^*n*_0_, and the number of photons detected by *D*_1_ is *rtn*_0_. For other *n* − *n*_0_ photons, Alice randomly controls *SW* according to her bit. Assume $p({a}_{j}^{(i)}={b}_{j}^{(i)})=p({a}_{j}^{(i)}\ne {b}_{j}^{(i)})=1/2$. The detection probability of each detector for these *n* − *n*_0_ states is shown in Table 1 and the clicks of the three detectors are listed in Table 2.

**Table 2:** Clicks of three detectors when Alice performs an intercept attack.

	Intercept *n*_0_	${\boldsymbol{n}}{\boldsymbol{-}}{{\boldsymbol{n}}}_{{\bf{0}}}{\boldsymbol{,}}{{\boldsymbol{a}}}_{{\boldsymbol{j}}}^{{\boldsymbol{(}}{\boldsymbol{i}}{\boldsymbol{)}}}{\boldsymbol{=}}{{\boldsymbol{b}}}_{{\boldsymbol{j}}}^{{\boldsymbol{(}}{\boldsymbol{i}}{\boldsymbol{)}}}$	${\boldsymbol{n}}{\boldsymbol{-}}{{\boldsymbol{n}}}_{{\bf{0}}}{\boldsymbol{,}}{{\boldsymbol{a}}}_{{\boldsymbol{j}}}^{{\boldsymbol{(}}{\boldsymbol{i}}{\boldsymbol{)}}}{\boldsymbol{\ne }}{{\boldsymbol{b}}}_{{\boldsymbol{j}}}^{{\boldsymbol{(}}{\boldsymbol{i}}{\boldsymbol{)}}}$
$N({\beta }_{0j}^{(i)}\mathrm{=1)}$	*r*^2^*n*_0_	*r*^2^(*n* − *n*_0_)/2	(*n* − *n*_0_)/2
$N({\beta }_{1j}^{(i)}\mathrm{=1)}$	*rtn*_0_	*rt*(*n* − *n*_0_)/2	0
$N({\alpha }_{j}^{(i)}\mathrm{=1)}$	*tn*_0_	*t*(*n* − *n*_0_)/2	0

Therefore, the total clicks for detectors *D*_0_, *D*_1_, and *D*_2_ are

17 $$\begin{array}{ccc}N({\beta }_{0j}^{(i)}=1) & = & {r}^{2}{n}_{0}+\frac{1}{2}{r}^{2}(n-{n}_{0})+\frac{1}{2}(n-{n}_{0})=\frac{5}{8}n-\frac{3}{8}{n}_{0};\\ N({\beta }_{1j}^{(i)}=1) & = & rt{n}_{0}+\frac{1}{2}rt(n-{n}_{0})=\frac{1}{8}n+\frac{1}{8}{n}_{0};\\ N({\alpha }_{j}^{(i)}=1) & = & t{n}_{0}+\frac{1}{2}t(n-{n}_{0})=\frac{1}{4}n+\frac{1}{4}{n}_{0}.\end{array}$$

When ${\alpha }_{j}^{(i)}=1$, Alice knows that Bob confirms her bit. Her optimal strategy is to alter one bit in the range of $n-N({\alpha }_{j}^{(i)}=1)$ bits. Among $n-N({\alpha }_{j}^{(i)}=1)$ bits, only $N({\beta }_{0j}^{(i)}=1)$ bits are not confirmed by Bob. Therefore, the probability that Alice alters one bit without detection by this attack is

18 $$p{\prime} (Aalter)=\frac{N({\beta }_{0j}^{(i)}\mathrm{=1)}}{n-N({\alpha }_{j}^{(i)}\mathrm{=1)}}=\frac{5n-3{n}_{0}}{6n-2{n}_{0}}\mathrm{}.$$

When Alice does not intercept, the probability of altering one bit without detection is *p*(*Aalter*) = 5/6. Since 0 < *p*′(*Aalter*) < 1 and *n*_0_ > 0, it can be see*n* that $p{\prime} (Aalter) < \frac{5n-3{n}_{0}}{6n-3{n}_{0}} < p(Aalter)$. A cheating Alice implementing an intercept attack has a larger probability of being detected by Bob than altered-straightway Alice. Then, this is not an effective attack.

#### Intercept/resend attack

When Alice performs an intercept attack, the numerator and denominator of *p*′(*Aalter*) are both decreased, and the decrease of the numerator is larger than that of the denominator. Then, it makes the successful attack probability even less than no interception and it is not an effective attack. Next, we analyze another similar attack, i.e., an intercept/resend attack. Alice controls the optical switch *SW* both at the times ${t}_{j}^{(i)}+\Delta {t}_{0}$ and ${t}_{j}^{(i)}+\Delta {t}_{1}$. When she detects each photon, she immediately sends another photon with the same polarization back to Bob’s site. This strategy can increase the clicks of detector *D*_0_, which may make Alice a larger probability of executing a successful attack. However, if Alice intercepts and resends all of the photons transmitted through the beam splitter *BS*, the numbers of photons detected by *D*_0_ and *D*_1_ are the same, which is different from the original ratio, and is detected by Bob. Therefore, Alice should select only a few photons and resend them back.

Assuming that Alice selects ${n{\prime} }_{0}$ bits to intercept and resend, for these ${n{\prime} }_{0}$ photons she controls the optical switch *SW* both at the times ${t}_{j}^{(i)}+\Delta {t}_{0}$ and ${t}_{j}^{(i)}+\Delta {t}_{1}$. Then, she resends the $t{n{\prime} }_{0}$ states back to Bob. The number of photons detected by *D*_2_ is $t{n{\prime} }_{0}$, the number of photons detected by *D*_0_ is ${r}^{2}{n{\prime} }_{0}+{t}^{2}{n{\prime} }_{0}$, and the number of photons detected by *D*_1_ is $2rt{n{\prime} }_{0}$. For other $n-{n{\prime} }_{0}$ photons, she randomly controls *SW* according to her bit. Assume that $p({a}_{j}^{(i)}={b}_{j}^{(i)})=p({a}_{j}^{(i)}\ne {b}_{j}^{(i)})=1/2$. The detection probability of each detector for these $n-{n{\prime} }_{0}$ states is shown in Table 1 and the clicks of the three detectors are listed in Table 3.

**Table 3:** Clicks of three detectors when Alice performs an intercept/resend attack.

	Intercept ${{\boldsymbol{n}}{\boldsymbol{{\prime} }}}_{{\bf{0}}}$	Resend ${{\boldsymbol{n}}{\boldsymbol{{\prime} }}}_{{\bf{0}}}$	${\boldsymbol{n}}{\boldsymbol{-}}{{\boldsymbol{n}}}_{{\bf{0}}}{\boldsymbol{,}}{{\boldsymbol{a}}}_{{\boldsymbol{j}}}^{{\boldsymbol{(}}{\boldsymbol{i}}{\boldsymbol{)}}}{\boldsymbol{=}}{{\boldsymbol{b}}}_{{\boldsymbol{j}}}^{{\boldsymbol{(}}{\boldsymbol{i}}{\boldsymbol{)}}}$	${\boldsymbol{n}}{\boldsymbol{-}}{{\boldsymbol{n}}{\boldsymbol{{\prime} }}}_{{\bf{0}}}{\boldsymbol{,}}{{\boldsymbol{a}}}_{{\boldsymbol{j}}}^{{\boldsymbol{(}}{\boldsymbol{i}}{\boldsymbol{)}}}{\boldsymbol{\ne }}{{\boldsymbol{b}}}_{{\boldsymbol{j}}}^{{\boldsymbol{(}}{\boldsymbol{i}}{\boldsymbol{)}}}$
$N({\beta }_{0j}^{(i)}\mathrm{=1)}$	${r}^{2}{n{\prime} }_{0}$	${t}^{2}{n{\prime} }_{0}$	${r}^{2}(n-{n{\prime} }_{0}\mathrm{)/2}$	$(n-{n{\prime} }_{0}\mathrm{)/2}$
$N({\beta }_{1j}^{(i)}\mathrm{=1)}$	$rt{n{\prime} }_{0}$	$rt{n{\prime} }_{0}$	$rt(n-{n{\prime} }_{0}\mathrm{)/2}$	0
$N({\alpha }_{j}^{(i)}\mathrm{=1)}$	$t{n{\prime} }_{0}$	0	$t(n-{n{\prime} }_{0}\mathrm{)/2}$	0

Therefore, the total clicks for detectors *D*_0_, *D*_1_, and *D*_2_ are

19 $$\begin{array}{ccc}N{\prime} ({\beta }_{0j}^{(i)}=1) & = & {r}^{2}{n{\prime} }_{0}+{t}^{2}{n{\prime} }_{0}+\frac{1}{2}{r}^{2}(n-{n{\prime} }_{0})+\frac{1}{2}(n-{n{\prime} }_{0})=\frac{5}{8}n-\frac{1}{8}{n{\prime} }_{0};\\ N{\prime} ({\beta }_{1j}^{(i)}=1) & = & rt{n{\prime} }_{0}+rt{n{\prime} }_{0}+\frac{1}{2}rt(n-{n{\prime} }_{0})=\frac{1}{8}n+\frac{3}{8}{n{\prime} }_{0};\\ N{\prime} ({\alpha }_{j}^{(i)}=1) & = & t{n{\prime} }_{0}+\frac{1}{2}t(n-{n{\prime} }_{0})=\frac{1}{4}n+\frac{1}{4}{n{\prime} }_{0}.\end{array}$$

Since Alice resends ${n{\prime} }_{0}$ photons, the total clicks are $N{\prime} ({\beta }_{0j}^{(i)}=1)+N{\prime} ({\beta }_{1j}^{(i)}=1)+N{\prime} ({\alpha }_{j}^{(i)}=1)=n+{n{\prime} }_{0}/2$. Among $N{\prime} ({\alpha }_{j}^{(i)}=1)$ bits, there are ${n{\prime} }_{0}/2$ bits intercepted and resent by Alice. The indexes of intercepted bits are the same as those of resent bits. According to Table 1, the bits that Alice knows that Bob confirms are exactly those detected by *D*_2_. For the intercepted ${n{\prime} }_{0}\mathrm{/2}$ bits, since the same indexes of states are resent back to Bob, Alice has no idea whether Bob confirms. For the resent ${n{\prime} }_{0}\mathrm{/2}$ bits, Alice cannot know whether Bob confirms either for the same reason. For the legal $n-{n{\prime} }_{0}$ bits, only $t(n-{n{\prime} }_{0}\mathrm{)/2}$ bits are detected by *D*_2_ and Alice knows Bob confirms. Therefore, when she alters one bit of a sequence, the altering range is also $n+{n{\prime} }_{0}/2-t(n-{n{\prime} }_{0})/2$. Only $N{\prime} ({\beta }_{0j}^{(i)}=1)$ bits are not confirmed by Bob, and Alice changing these bits would not be detected. Therefore, the probability that Alice alters one bit without detection by this attack is

20 $$p{\prime\prime} (Aalter)=\frac{N{\prime} ({\beta }_{0j}^{(i)}=1)}{n+{n{\prime} }_{0}/2-t(n-{n{\prime} }_{0})/2}=\frac{5n-{n{\prime} }_{0}}{6n+6{n}_{0}}.$$

It can be seen that *p*″(*Aalter*) < *p*(*Aalter*). The intercept/resend attack makes Alice being detected by Bob a larger probability, and it is not an effective attack either.

#### Reflection attack

There is another special intercept/resend attack, denoted as reflection attack. In this attack, Alice does not choose her bit in the *commit phase* for each bit. When Alice does nothing with the switch *SW*, the setup constitutes a Michelson interferometer and *D*_0_ clicks. In this case, Bob has no idea about Alice’s bit. Then Alice can lie about her value, while Bob has zero probability to detect it. However, the verification in Step 6 makes this attack fail. The security analysis of the attack is as follows.

For each N-bit string *a*^(*i*)^, Alice follows the protocol honestly for (*N* − *l*) bits, except for *l* bits. *l* is a tiny percentage of *N*, usually is one or several bits. Otherwise, the cheating will make the incorrect clicks of the detector *D*_0_. If all of *l* bits are chosen to be the verified bits, the reflection attack does not work. The probability that Alice alters one bit without detection in one sequence is

21 $${p}_{1}(Ref)=\frac{{C}_{k}^{l}}{{C}_{N}^{l}}\cdot p(Aalter)=\frac{5}{6}\cdot \frac{{C}_{k}^{l}}{{C}_{N}^{l}}.$$

If there are at least one bit in the remain *N* − *k* bits, Alice’s reflection attack cannot be detected, the probability of which is

22 $${p}_{2}(Ref)=1-\frac{{C}_{k}^{l}}{{C}_{N}^{l}}.$$

Then, when Alice applies the reflection attack in one sequence, the probability of the successful cheating is

23 $$p(Ref)={p}_{1}(Ref)+{p}_{2}(Ref)=1-\frac{{C}_{k}^{l}}{6{C}_{N}^{l}}.$$

The probability of Alice changing the committed bit without detection is *p*(*Ref*)^*m*/2^. It can be seen that a larger *k* leads a more secure protocol. Although 5/6 < *Pr*(*RA*) and the reflection attack is more efficient than changing the bit directly, it satisfies *Pr*(*RA*) < 1, the protocol can satisfy the binding security by choosing a larger security parameter *m* to make *p*(*Ref*)^*m*/2^ be exponentially small.

#### No-go-theorem attack

The framework of the no-go theorem is described as follows^6^.

1. Alice chooses the committed bit $x\in \mathrm{\{0,1\}}$ and she prepares the state
  24 $$|x\rangle =\sum _{i}\,{\alpha }_{i}^{(x)}|{e}_{i}^{(x)}{\rangle }_{A}\otimes |{\phi }_{i}^{(x)}{\rangle }_{B},$$
  where ${\langle {e}_{i}^{(x)}|{e}_{j}^{(x)}\rangle }_{A}={\delta }_{ij}$ while $|{\phi }_{i}^{(x)}{\rangle }_{B}$’s are not necessarily orthogonal to each other.
2. An honest Alice is supposed to make a measurement on the register A and determine the value of *i*.
3. Alice sends the second register B to Bob as a piece of evidence for the commitment.
4. In the unveil phase, Alice opens the commitment by declaring the values of *x* and *i*.
5. Bob performs measurements on the register B to verify whether Alice’s declared data are correlated with his measurement results.
6. To ensure the concealing of the QBC protocol, the density matrices describing the second register are approximative, i.e.,
  25 $$T{r}_{A}|0\rangle \langle 0|\equiv {\rho }_{0}^{B}\simeq {\rho }_{1}^{B}\equiv T{r}_{A}|1\rangle \langle 1|.$$
7. Cheating strategy. When Eq. (25) is satisfied, Alice can apply a local unitary transformation to rotate $\mathrm{|0}\rangle$ to $\mathrm{|1}\rangle$ without detection.

In the SCQBC protocol, the quantum states are prepared by Bob, and Alice has no initial states. If Alice wants to attack using the no-go theorem, she tries to perform a controlled unitary transformation instead of the protocol operation, which is inspired by^33^. The control bit in the transformation is entangled with the other register; that is, when Alice commits “0”, the entire state is

26 $$\begin{array}{rcl}\mathrm{|0}\rangle  & = & \frac{1}{{2}^{n-1}}\sum _{{a}_{1}^{(i)}\oplus \ldots \oplus {a}_{n}^{(i)}\mathrm{=0}}\,|{a}_{1}^{(i)}\ldots {a}_{n}^{(i)}{\rangle }_{A}{U}_{B}({a}_{1}^{(i)}\ldots {a}_{n}^{(i)})\underset{j=1}{\overset{n}{\otimes }}|{\Psi }_{{b}_{j}^{(i)}}{\rangle }_{B}\\  & = & \frac{1}{{2}^{n-1}}\sum _{{a}_{1}^{(i)}\oplus \mathrm{..}.\oplus {a}_{n}^{(i)}\mathrm{=0}}\,|{a}_{1}^{(i)}\ldots {a}_{n}^{(i)}{\rangle }_{A}{[{U}_{B}({a}_{1}^{(i)})|{\Psi }_{{b}_{1}^{(i)}}\rangle }_{B}]\otimes \ldots \otimes {[{U}_{B}({a}_{n}^{(i)})|{\Psi }_{{b}_{n}^{(i)}}\rangle }_{B}]\\  & = & \frac{1}{{2}^{n-1}}\sum _{{a}_{1}^{(i)}\oplus \mathrm{..}.\oplus {a}_{n}^{(i)}\mathrm{=0}}\,|{a}_{1}^{(i)}\ldots {a}_{n}^{(i)}{\rangle }_{A}|{\Psi {\prime} }_{{b}_{1}^{(i)}}{\rangle }_{B}\otimes \ldots \otimes |{\Psi {\prime} }_{{b}_{n}^{(i)}}{\rangle }_{B}\mathrm{}.\end{array}$$

Similarly, when Alice commits “1” the entire state is

27 $$|1\rangle =\frac{1}{{2}^{n-1}}\sum _{{a}_{1}^{(i)}\oplus \ldots \oplus {a}_{n}^{(i)}=1}\,|{a}_{1}^{(i)}\ldots {a}_{n}^{(i)}{\rangle }_{A}|{\Psi {\prime} }_{{b}_{1}^{(i)}}{\rangle }_{B}\otimes \ldots \otimes |{\Psi {\prime} }_{{b}_{1}^{(i)}}{\rangle }_{B}.$$

Since the concealing of SCQBC protocol can be satisfied, Alice may perform a local unitary transformation to rotate $\mathrm{|0}\rangle$ to $\mathrm{|1}\rangle$. However, two characters limit this attack and it cannot work.

1. In the SCQBC protocol, the operation of Alice is to control the optical switch *SW* at different times according to ${a}_{j}^{(i)}$. The *SW* is a macroscopic device that Alice’s operation cannot treat as $|{a}_{j}^{(i)}{\rangle }_{A}$ or other quantum states. The problem is the entanglement of macroscopical devices and microscopical states, which is hardly applied at present, like “Schroedinger’s cat.” Moreover, there need to be exponential quantum states entangled with the macroscopic devices to apply a no-go-theorem attack. How to realize the entanglement and whether it is possible to realize it have not been determined.
2. Even if Alice can make her local quantum register entangle with Bob’s qubits, a no-go-theorem attack would not be realized. Analyzing the counterfactual-kind quantum protocol from the perspective of the particle-like nature of light, half of the particles do not arrive at Alice’s site. Therefore, Alice cannot perform the local controlled unitary transformation with all the n-qubit states. Analyzing the counterfactual-kind quantum protocol from the perspective of the wave character of light, a state sent by Bob is formed by an *a* mode and *b* mode, where the wave packet in *a* mode is in Bob’s site and the wave packet in *b* mode transfers to Alice’s site. Alice, at best, makes her registers entangle with the packets in *b* mode, which cannot rotate the entire state to change the commitment without detection. The pre-condition of a no-go-theorem attack is to make all the commitment-related qubits entangled with all of Bob’s qubits. Alice cannot access Bob’s evidence in the SCQBC protocol, and therefore she cannot apply a no-go-theorem attack.
3. In ideal SCQBC, Bob sends the single-photon states. The photons are either detected by Bob or by Alice. Once Alice obtains the states sent by Bob and wants to use them to attack, Bob knows her choice and she cannot change the bit anymore, even if a stronger no-go-theorem attack exists.

## Discussion

There is a general framework of bit commitment protocol extracted from the SCQBC protocol, which is described as follows.

### Protocol 2 Framework of bit commitment protocol

#### Commit phase

1. *Alice and Bob agree on two security parameters* *m* *and* *n*.
2. *Alice chooses a random bit* $x\in \mathrm{\{0,}\,\mathrm{1\}}$ *as her committed bit. Then, she generates* *m* *random bit strings according to the value of* *x*. *Each sequence consists of* *n* *bits, which can be represented as* ${a}^{(i)}\equiv ({a}_{1}^{(i)}{a}_{2}^{(i)}\mathrm{..}.{a}_{n}^{(i)})\in {\{0,1\}}^{n}$, $i=\mathrm{1,}\,\mathrm{2,}\,\mathrm{...,}\,m$. *Each sequence satisfies* ${a}_{1}^{(i)}\oplus {a}_{2}^{(i)}\oplus \mathrm{.}..\oplus {a}_{n}^{(i)}=x$.
3. *Bob generates m bit strings randomly and uniformly with the length of n. Each sequence is represented as* ${b}^{(i)}\equiv ({b}_{1}^{(i)}{b}_{2}^{(i)}\mathrm{..}.{b}_{n}^{(i)})\in {\{0,1\}}^{n}$.
4. *Alice and Bob invoke another particular bit-comparison function module as a black box to give some evidence of commitment to Bob. In this step, Bob compares* ${b}_{j}^{(i)}$ *with* ${a}_{j}^{(i)}$ *bit-by-bit and knows* ${b}_{j}^{(i)}={a}_{j}^{(i)}$, ${b}_{j}^{(i)}\ne {a}_{j}^{(i)}$, *or nothing. For each bit-comparison, Bob can confirm the value of Alice’s bit with a probability p and Alice knows that Bob confirms her bit with a probability q, where* 0 ≤ *q* < *p* < 1.

#### Unveil phase

1. *Alice reveals the value of x and m sequences* $({a}_{1}^{(i)}{a}_{2}^{(i)}\ldots {a}_{n}^{(i)})$, *i* = 1, 2, …, *m to Bob*.
2. *From the particular bit-comparison scheme, Bob knows some of Alice’s bits. Therefore, Bob can verify whether Alice’s opening results are consistent with the bits he knows. Then, he needs to verify whether* ${a}_{1}^{(i)}\oplus {a}_{2}^{(i)}\oplus \mathrm{.}..\oplus {a}_{n}^{(i)}=x$. *If the consistency of all the data holds, he admits Alice’s commitment value as x; otherwise, if any of the data are inconsistent, he concludes that Alice cheated*.

In Step 4, there is a bit comparison black box. In the SCQBC protocol, the black box is realized by the counterfactual setup. The complete security proof of the BC framework and other constructions of the black box are left for future study. However, the framework may open up a new idea for unconditionally secure BC protocols.

## Conclusions

In this paper, we construct a SCQBC protocol inspired by counterfactual quantum cryptography. We then analyze the binding and concealing security of the protocol. For concealing security, we point that a cheating Bob sending illegal states and using single-illegal device (*BS*) can be detected by Alice. Specially, when Bob employs several illegal devices, although the attack seems works in theory, the physical extremes of the device makes there always exists a secure parameter *N* to guarantee the concealing. For binding security, we point that Alice’s intercept attack and intercept/resend attack are both ineffective attacks. There are three characteristics of the SCQBC protocol, as follows. (i) The optical switch *SW* is a macroscopic device and Alice’s operation cannot be controlled by quantum states. (ii) For the counterfactual-type quantum protocol, some information does not get transmitted through the channel and Alice does not have enough information to apply a no-go-theorem attack. (iii) Once Alice obtains the states sent by Bob and wants to use them to execute a no-go-theorem attack, Bob knows her choice and she can no longer change the bit. These characteristics guarantee that Alice cannot apply a no-go theorem attack. Although SCQBC protocol have not been proved to be unconditionally secure, it remains secure in practice against the general attacks and the no-go theorem type attacks so far. And SCQBC is an extension of counterfactual quantum cryptography and may stir great interest in QBC research.
